# A new method to localise and quantify oxidative stress in live juvenile mussels

**DOI:** 10.1242/bio.059030

**Published:** 2021-12-14

**Authors:** Natalí J. Delorme, Alfonso J. Schmidt, Leonardo N. Zamora, David J. Burritt, Norman L. C. Ragg

**Affiliations:** 1 Aquaculture Group, Cawthron Institute, Private Bag 2, Nelson 7042, New Zealand; 2 Hugh Green Cytometry Centre, Malaghan Institute of Medical Research, PO Box 7060, Wellington 6242, New Zealand; 3 Botany Department, University of Otago, PO Box 56, Dunedin 9054, New Zealand

**Keywords:** Reactive oxygen species, Greenshell™ mussel, Aquaculture, Spat, Emersion stress, Fluorescent staining

## Abstract

Stress and survival of the juvenile New Zealand green-lipped mussel, *Perna canaliculus*, is a poorly understood bottleneck in the ecological and economic performance of a significant aquaculture crop. This species was therefore selected as a model organism for the development of a new method to quantify oxidative stress in whole individuals. An *in vivo* ROS-activated stain (CellROX™) was administered to anaesthetised, translucent juveniles that were subsequently formaldehyde fixed and then visualised using confocal microscopy. Subsequent application of image analysis to quantifying ROS-positive tissue areas was successfully used to detect stress differences in juvenile mussels exposed to varying levels of emersion. This integrated method can be used to localise and quantify ROS production in individual translucent bivalve life stages (larval and juvenile), while relative stability following fixation greatly expands potential practical field applications.

This article has an associated First Person interview with the first and third authors of the paper.

## Abstract (precis)

**Summary:** A new method to quantify reactive oxygen species in live mussels using image analysis. The unusual fixation step allows for *in situ* staining, followed by image analysis in the laboratory.

## Introduction

Reactive oxygen species (ROS) are highly reactive, and potentially damaging, derivatives of molecular oxygen (Berg et al., 2019). Examples of ROS include hydrogen peroxide (H_2_O_2_), superoxide anion radical (O_2_^−^), alkoxyl radical (RO), peroxyl radicals (ROO), singlet oxygen (^1^O_2_) and hydroxyl radical (OH), among others (Alves de Almeida and Di Mascio, 2011; González et al., 2015; Phaniendra et al., 2015). ROS are constantly produced during normal cell respiration, but are typically neutralised by the action of enzymatic and non-enzymatic antioxidant defences naturally found in healthy cells (Espinosa-Diez et al., 2015; Burritt and Lamare, 2016). In healthy organisms, there is a balance between pro-oxidants and antioxidants, with approximately 1-3% of the consumed oxygen in molluscs being converted to ROS (Lewis and Santos, 2016). Even though ROS are considered hazardous species for the organism, ROS also have an important role in cellular signalling and in the maintenance of homeostatic conditions (González et al., 2015; Gomes et al., 2005). The natural balance between ROS and antioxidants can be disrupted under stressful conditions, where ROS accumulate within the cells, resulting in oxidative stress (Berg et al., 2019). Stressful conditions can be caused by abiotic (e.g. environmental pollutants) or biotic (e.g. pathogens) factors, with the accumulation of ROS resulting in damage to key macromolecules (DNA, lipids and proteins), consequently affecting the overall performance of an organism and potentially compromising its survival.

There are several fluorescence probes available to detect different ROS (Gomes et al., 2005). The most widely used probes in shellfish research are 2′,7′-dichlorodihydrofluorescein diacetate (DCFH_2_-DA) which detects mainly H_2_O_2_ but has also been shown to detect OH^.^ and ROO^.^, and dihydroethidium (DHE) which specifically detects O_2_^−^ (Gomes et al., 2005; Rivera-Ingraham et al., 2016, 2013; Delorme et al., 2021b; Rolton et al., 2020; Rolton and Ragg, 2020; Donaghy et al., 2012; Nguyen and Alfaro, 2019). Despite the wide range of probes design to detect ROS in biological samples, the vast majority do not allow for fixation of the samples prior to analysis, which limits the practical use of these probes. However, recent proprietary probes such as CellROX^TM^, which detect general ROS in live cells, allow for fixation of the samples, expanding the potential use of ROS detection probes.

The green-lipped mussel, *Perna canaliculus* (Gmelin, 1791), also known as the Greenshell™ mussel, is an important foundation species and one of the most significant aquaculture crops in New Zealand (Stenton-Dozey et al., 2020). Juveniles of this species are predominantly collected from the wild, representing around 80% of the total ‘seed’ used by the aquaculture industry, with the remainder being produced in hatcheries (Jeffs et al., 2018; Delorme et al., 2019; South et al., 2022). Regardless of the source, the seed must be transported to marine farming locations for grow-out, a transfer which typically takes 20-72 h (South et al., 2020b). Retention of mussels after seeding is currently one of the bottlenecks of the industry, with losses of around 85% of mussels within the first 90 days (South et al., 2020a). Currently, it is uncertain whether mussel losses are related to the natural detachment behaviour of the mussels (secondary settlement) (South et al., 2020a), the poor quality of the mussels being seeded (Sim-Smith and Jeffs, 2011), or mortality due to stressful transport and/or farm conditions (Supono et al., 2020; Delorme et al., 2019).

Currently, the methods used to assess the condition of juvenile *P. canaliculus* are limited to fitness tests using Fast Green dye (Webb and Heasman, 2006), or measurement of glycogen reserves (Sim-Smith and Jeffs, 2011) or gut contents (Delorme, 2018). Methods that directly quantify stress levels are required to assess the physiological condition of juvenile mussels to support husbandry refinement trials and provide a general ecophysiological monitoring tool. Recent work has highlighted the importance of oxidative damage to macromolecules in *P. canaliculus* juveniles experiencing environmental stress (Delorme et al., 2020), highlighting the need for effective quantification of ROS. Therefore, in the present study we used *P. canaliculus* as a model organism for the development of a new method to quantify ROS levels in small, translucent juveniles, that combined physiological staining, confocal microscopy and quantitative image analysis.

## RESULTS AND DISCUSSION

Probes to detect and quantify reactive oxygen species (ROS) are typically used in isolated cells or tissues. In shellfish, previous studies have quantified ROS in extracted haemolymph (e.g. Delorme et al., 2021b, Rolton and Ragg, 2020) or excised gill tissue (e.g. Rivera-Ingraham et al., 2016). The present study shows a novel technique that was developed to determine and quantify ROS in individual living shellfish. The protocol is appropriate for translucent juvenile mussels that do not have a heavily calcified or coloured shell (up to ∼4-5 mm of shell length in *Perna canaliculus*). This protocol could also be used in response to other biotic or abiotic stressors, e.g. hypoxia-reoxygenation stress, temperature fluctuations, bacterial infections, algal toxins.

Pilot trials using *P. canaliculus* determined that a standard staining incubation time of 30 min is sufficient to successfully detect and quantify ROS in haemocytes (Delorme et al., 2021b; Rolton and Ragg, 2020). However, when assessing *P. canaliculus* veliger larvae, increasing incubation time to 1 h resulted in an improved fluorescent signal (Delorme, unpublished data). Juvenile *P. canaliculus* typically closed their valves immediately after addition of the CellROX™ Green dye, reducing tissue exposure and further limiting the fluorescent signal. A non-toxic anaesthetic applied prior to staining and extending to 1 h the incubation time is therefore recommended for bivalves that can reactively isolate from their surrounding environment by closing their shells.

The distribution of the fluorogenic ROS signal observed inside the juvenile mussels shows that a larger part of the signal is located in the mussel mantle tissue. The function of the mantle in molluscs is to form the shell by secreting and depositing conchiolin and calcium carbonate as mussels grow (Chapman and Barker, 1964). Therefore, it is possible that a higher ROS signal in the mantle of juvenile mussels is the result of a higher metabolic activity of the mantle epithelial cells associated with shell formation in actively growing juveniles. Additionally, fluorescence intensity may depend on the volume or mass of the stained tissue. Using a different approach in which the quantification of the ROS signal is standardized by tissue volume or mass could perhaps result in a better understanding on how different tissues are being specifically impacted by the applied stress.

**Fig. 1.**
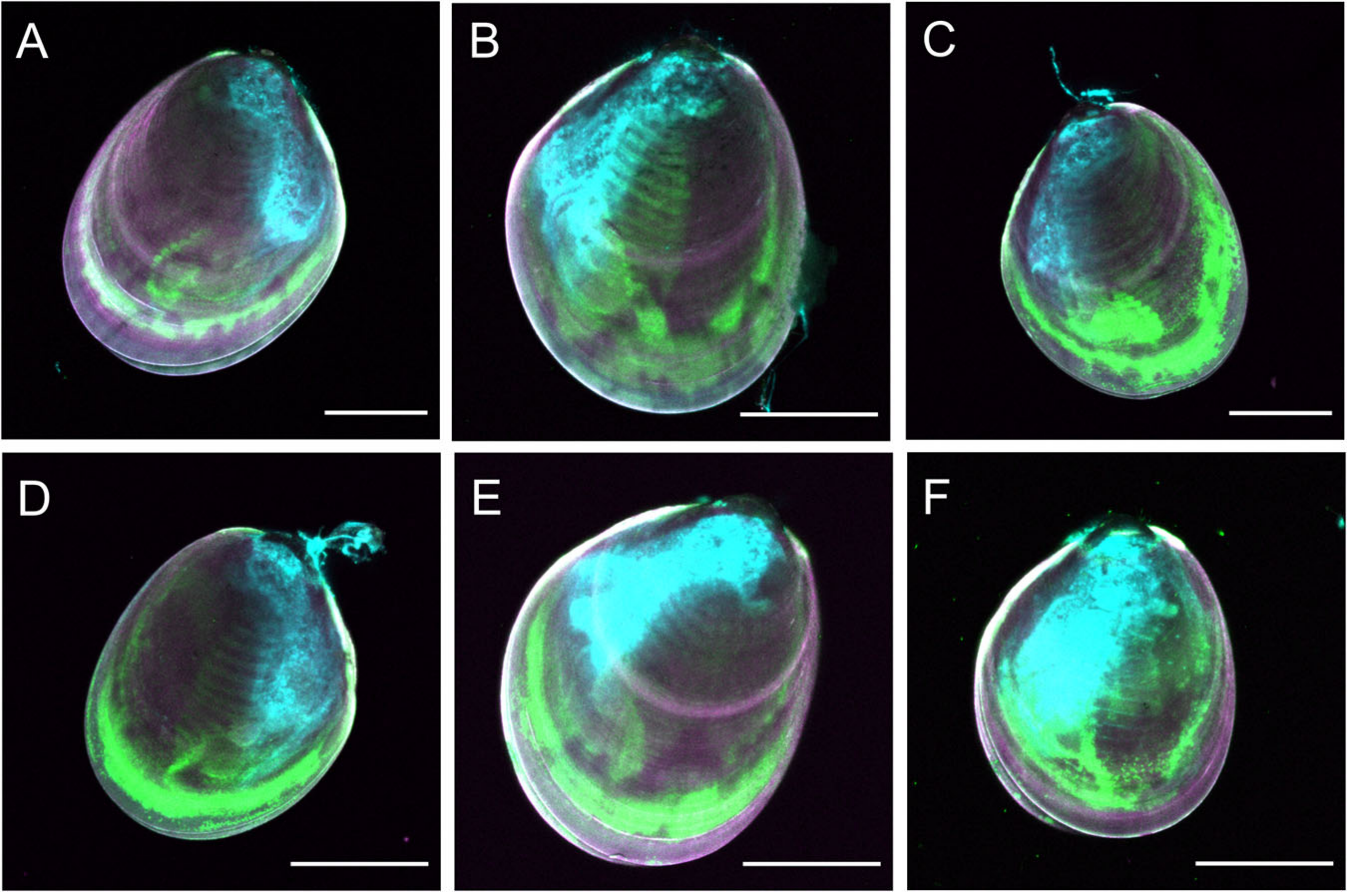
**Fluorogenic signal of ROS in juvenile *P. canaliculus* exposed to emersion durations (1 and 20 h) at different relative humidity (RH) levels (high and moderate).** (A) Control (mussels always immersed in seawater); (B) 1 h emersion at high RH (1 h-H); (C) 1 h emersion at moderate RH (1 h-M); (D) 20 h emersion at high RH (20 h-H); (E) 20 h emersion at moderate RH (20 h-M); (F) Dead mussels that were killed by freezing at −20°C prior to the staining and fixing. ROS signal was detected using the probe CellROX™ Green. Mussels were treated with magnesium chloride (MgCl_2_) as an anaesthetic prior to performing the ROS staining protocol. Magenta: shell autofluorescence (blue channel, CH1); green: ROS signal (green channel, CH2); cyan/turquoise: gut autofluorescence (red channel, CH3). Scale bars: 500 µm.

**Fig. 2.**
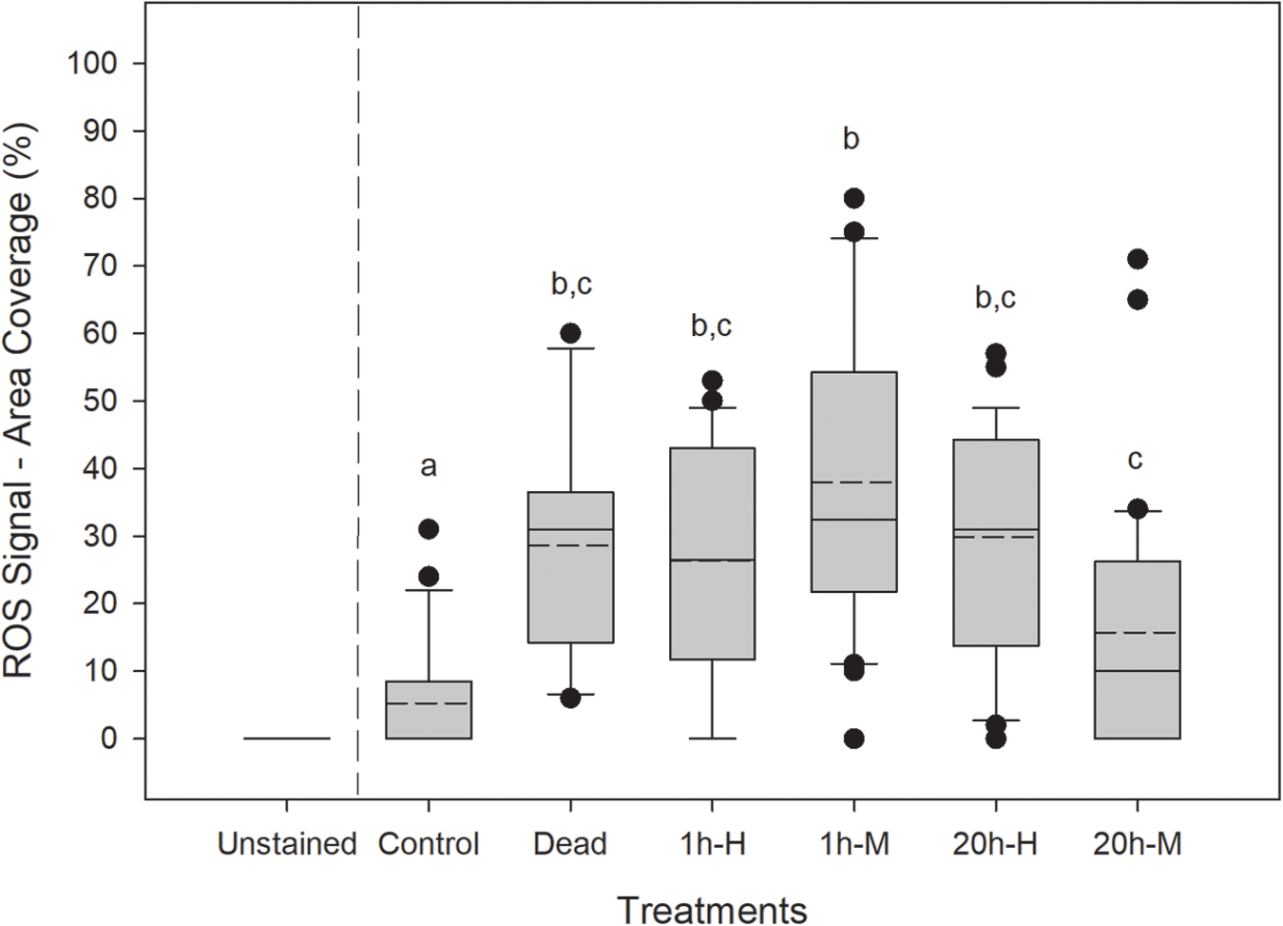
**Boxplot showing the variability of the data for ROS signal in juvenile *P. canaliculus* exposed to air (1 and 20 h) at different relative humidity (RH) levels (high and moderate).** Unstained: negative staining control (NSC) for imaging; control: mussels immersed in seawater; dead: mussels killed by freezing at −20°C overnight prior to the staining and fixing procedure; 1 h-H: 1 h emersion at high RH; 1h-M: 1 h emersion at moderate RH; 20 h-H: 20 h emersion at high RH; 20 h-M: 20 h emersion at moderate RH. The median and mean values for each box are shown with solid and dashed lines, respectively (*n*=10 for each treatment). Unstained group was excluded from the statistical analysis as they were used as a negative staining control for imaging only. Significant differences (*P*<0.05) among groups are denoted by different lower-case letter above bars.

Juvenile *P. canaliculus* can be stranded by the receding tide or exposed to air during transfer from the hatchery to grow-out facilities in aquaculture (Delorme et al., 2020; Jeffs et al., 2018). Additionally, emersion in juvenile *P. canaliculus* has shown to result in high oxidative damage, particularly after 1 h of rehydration after emersion (Delorme et al., 2021a). Hence, the present study applied emersion and relative humidity treatments as relevant stressors likely to induce a range of ROS levels in *P. canaliculus* juveniles (Figs 1 and 2). Here, emersion and relative humidity had a significant effect on the ROS signal quantified in juvenile mussels (nested analysis of variance, ANOVA, *F*_8,154_=8.391, *P*=0.0048). Mussels that did not experience an emersion and relative humidity stress show significantly lower ROS signal than the other treatment groups (Fig. 2). There were no significant differences in ROS signal when juvenile mussels were exposed to air at high RH (Tukey, *P*=0.985; Fig. 2). However, when mussels were exposed to moderate RH during emersion, the ROS signal was *ca*. 60% higher in mussels that experienced emersion for 1 h than mussels exposed to air for 20 h (Tukey, *P*<0.001; Fig. 2). This response was unexpected and could be explained by homeostatic stress response activating antioxidant mechanisms to neutralise ROS in the organism (Espinosa-Diez et al., 2015). Since only living, apparently vigorous juvenile mussels were selected for staining after treatment, it is possible that under prolonged emersion conditions the antioxidant mechanisms in the selected mussels overcompensated for the large accumulation of ROS, resulting in a relatively low ROS signal (mean of 16% ROS) compared with the other treatments (mean between 26-38% ROS), but still higher than control mussels (mean of 5% ROS). Previous research has shown that the antioxidant activity increases greatly in juvenile *P. canaliculus* after exposure to different emersion times and relative humidity levels, especially when mussels are exposed to mid humidity conditions (Delorme et al., 2021a). This result suggests that selected (living) mussels were able to cope with the prolonged stress caused by a 20 h emersion and 1 h of recovery in seawater, but their subsequent capacity to completely recover and survive in the long term remains unknown. Recent studies in juvenile *P. canaliculus* have shown that survival during recovery (up to 10 h) is greatly compromised as relative humidity decreases (Delorme et al., 2021a). It is also worth noting that the strength of the ROS signal observed in dead mussels was comparable to that of stressed mussels. This may be attributable to the cold shock that the mussels suffered during the freezing, inducing ROS production in the process. The ROS signal found in dead (freeze-killed) mussels indicates that the ROS dye used in this study can penetrate dead cells and tissues and can be oxidised by the presence of preformed ROS or other oxidants. It is also important to note that oxidation reactions can still occur in dead tissues (Domínguez et al., 2019), and that some of these oxidation products could react with fluorogenic probes to give a fluorogenic signal. Therefore, it may be possible that a fluorogenic ROS signal could still be visible if mussels died during exposure to a stressor, but the production and lifespan of fluorogenic ROS signals in dead or frozen, and then thawed, tissues require further investigation.

In this study, juvenile mussels that were exposed to 1 h of emersion at moderate RH showed an increase of *ca*. 30% in ROS signal compared to mussels experiencing 1 h emersion at high RH (Tukey, *P*<0.022; Fig. 2). The opposite trend was observed when mussels were exposed to 20 h of emersion, where mussels experiencing high RH had a *ca*. 48% higher ROS signal compared to those that experienced 20 h of emersion at moderate RH (Tukey, *P*=0.001; Fig. 2). The differential ROS signal between treatments may be explained by differences in behaviour between mussels maintained under different conditions during emersion. It is possible that mussels opened their valves during emersion while others closed their valves, as observed in adult mussels showing different gaping behavioural strategies in response to emersion (Powell et al., 2017; Zamora et al., 2019). Mussels closing their valves would be potentially under anaerobic metabolism, presumably reducing ROS production compared to mussels attempting to maintain aerobic respiration under emersion conditions.

Overall, this study showed that the ROS detection protocol performed on juvenile *P. canaliculus* effectively distinguished differences in environmentally induced stress levels through quantification of the ROS signal in terms of area of coverage. While emersion stress in juvenile *P. canaliculus* has previously been shown to result in the accumulation of oxidative damage markers (Delorme et al., 2019, 2021a), as a consequence of increased ROS in the cells (Freire et al., 2012), the direct ROS quantification presented in the present study reveals complex kinetics that warrant further investigation. Additionally, future research would also benefit from exploring alternative fluorogenic probes to gain more insight to the origins of ROS production. Probes that detect different ROS types could be used to identify tissue-specific ROS production as previously seen in bivalve gill tissue (Rivera-Ingraham et al., 2016). Other probes that target mitochondrial ROS could be used to better understand the origins of ROS production under different stressful conditions. Furthermore, probes that detect either the effects of ROS accumulation in the cells, such as lipid peroxidation probes, or fluorogenic probes that detect antioxidants such as glutathione (indicating the ability of the cell to prevent oxidative stress) would complement this research and would allow us to better understand the fundamentals of the redox biology of juvenile *P. canaliculus*.

## Conclusions and Recommendations

The method presented here allow variability in ROS signals to be observed within each replicate sample, detecting different levels of stress in juvenile *P. canaliculus*. While substantial inter-individual variability was observed (Fig. 2), a sample size of 10 random individuals from each replicate sample was sufficient to detect significant statistical differences among treatments. In this study, the 10 individuals were photographed separately, however, the method could be modified to obtain single picture that includes multiple individuals, which would reduce labour and cost associated with performing the staining method presented here.

**Fig. 3.**
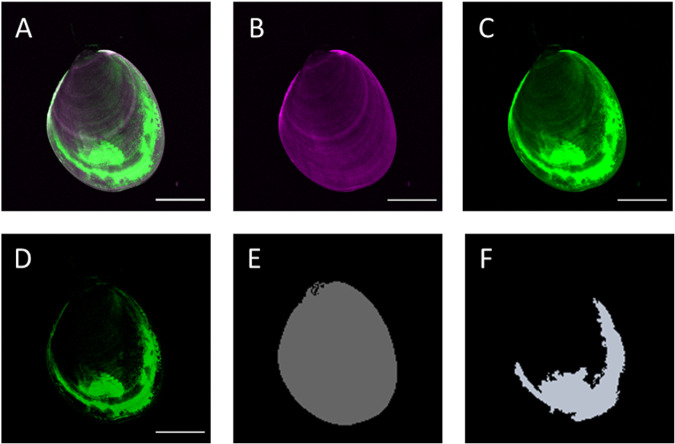
**Schematic image analysis pipeline used to quantify ROS signal in juvenile *P. canaliculus*.** (A) Maximum intensity projection shell autofluorescence (magenta) and ROS positive signal (green); (B) fluorogenic signal from blue channel (CH1); (C) fluorogenic signal from green channel (CH2); (D) result from subtraction of signal from green and blue channels; (E) shell area selection of the mussel; (F) ROS positive area selection. Scale bars: 500 µm.

Fluorescent signal intensity was observed to vary with the level of stress in the mussels. For example, fluorescent intensity in 1h-M mussels was higher than the other treatments, and 20h-M intensity was low, matching the calculated area, but intensity for this treatment was very variable (results not shown). Future studies could therefore productively focus on investigating methods to include signal intensity as a parameter for quantification of ROS as well as having other measures of stress as survival. ROS signal was also mainly detected in mantle, with a lower signal observed in the gills and visceral mass. These tissue-specific differences in ROS signal should be studied further to understand better how different stressors affect the redox biology in mussel juveniles. The method presented here is an end-point assay, and fixation is part of the methodology to broaden the application of the method, however, this method could also be used without the fixation step and be analysed in real time in order to understand the kinetics of ROS production during and after the stress has been applied to the mussels. A composite approach for image analysis could also be developed to identify other relevant signals within the mussels, such as gut content, as shown in this study by using CH3 (the red channel; Fig. 3). The use of multiple probes and/or stains to determine a variety of physiological responses in juvenile shellfish would broaden the applications of the methodology presented here, which could also be adapted for high throughput assays using a plate-reader or high content screening (HCS) technology.

The method for localisation and quantification of ROS developed in this study has potential applications for the aquaculture industry, where quality of the juvenile mussels that are seeded onto the farms could be tested. In general, most oxidative stress staining protocols use probes that cannot be fixed, limiting the potential applications of these methods outside a laboratory setting. The fact that the protocol developed in this study includes a fixation step introduces significant flexibility for sample collection, where staining could be performed *in situ,* either in the field or commercial hatcheries, and the samples sent to a laboratory for imaging and analysis within 24 h of sample collection and staining.

## MATERIALS AND METHODS

Juvenile *P. canaliculus* were sourced from a commercial hatchery (SPATnz) and transported to the adjacent Cawthron Aquaculture Park (Nelson, New Zealand). The juveniles had recently been screened to ensure that a homogenous size range (1-1.5 mm shell length) were used for experimentation. These juveniles were still translucent (no shell colouration).

The probe used to detect and measure oxidative stress in mussels was CellROX™ Green (Invitrogen™). The typical application of this reagent is to detect and quantify reactive oxygen species (ROS) in live cells. This reagent is cell-permeant and exhibits a bright green, photostable fluorescence upon oxidation by ROS and subsequent binding to DNA. Signal has maximum excitation and emission at 485 and 520 nm, respectively. Cells can be fixed using formaldehyde and the fluorogenic signal read within 24 h. Stock concentration of the reagent (2.5 mM) were prepared in DMSO (dimethylsulfoxide) and a working solution (WS) was prepared to achieve 100 µM of the reagent in DMSO (hereafter called ROS WS). Aliquots of the ROS WS were stored in the dark at −20°C until use.

### Detecting different levels of emersion stress in juvenile mussels

This experiment was designed to validate the staining protocol by creating different levels of stress in juvenile mussels exposed to air (1 or 20 h) and two relative humidity (RH) treatments (high: 85% and moderate: 60%). Emersion treatments were run in cylindrical 750 ml air-tight plastic containers with either a 4×4 cm wet cotton cloth to maintain a high RH or 1.5 g of silica gel to achieve a moderate RH (Delorme et al., 2021a). Containers were assigned to the two RH treatments (six containers each), which were further assigned to two emersion periods (three containers each). RH loggers were added to the containers allocated to 20 h of emersion.

Juvenile mussels were placed in the six containers assigned to the 20 h emersion treatment (∼100 mg mussels in each container) and maintained at 20°C overnight. Control mussels were incubated in a 500 ml glass beaker containing aerated, 0.35 µm filtered seawater (FSW). A sub-sample of ∼20 mussels was stored overnight at −20°C to create a dead staining reference. Mussels were subsequently added to the 1 h emersion treatment, synchronising the end of the challenge period. The damp cloth or silica gel was removed and FSW added to each container for 1 h of rehydration. Then, ∼20 mussels from each container were transferred into 1.7 ml microcentrifuge tubes; triplicate subsamples were collected from the control beaker. Only living mussels were sampled for staining (mussels that showed foot movement). An extra sample tube of mussels was collected to be used as a negative staining control (NSC) for imaging.

Preliminary tests indicated that juvenile mussels needed to be anaesthetised to induce opening of the valves to facilitate the staining process and ensure all mussels have the same exposure time to the dye. Magnesium chloride (MgCl_2_) was used as an anaesthetic as this has been used as a non-toxic anaesthetic in several marine species (e.g. Alipia et al., 2014, Arafa et al., 2007, Pugliese et al., 2016). The preliminary test showed that ROS signal in unstressed juvenile *P. canaliculus* is not affected by the addition of MgCl_2_ (ROS% without MgCl_2_±s.d.=2.69±1.07%; ROS% with MgCl_2_±s.d.=2.61±1.14%; Student's *t*-test: t=0.122, d.f.=6, *P*=0.907); which indicates that MgCl_2_ does not induce ROS production in juvenile *P. canaliculus*. Sample tubes of mussels, including the NSC controls, were therefore treated with 3.75% magnesium chloride in seawater for 30 min. This incubation time was sufficient to induce valve opening in the mussels. After incubation, the mussels were rinsed three times with FSW; 190 µl of FSW was then added to the tubes from each treatment and to the freeze-killed sub-sample, while 200 µl of FSW were added to the NSC tube.

For staining, 10 µl of the ROS WS were added to each tube to achieve a final concentration of 5 µM. No ROS WS was added to the NSC. Tubes were incubated in the dark at room temperature (20°C) for 1 h, following pilot trials that indicated that the manufacturer's recommended 30 min incubation resulted in a weak ROS fluorescent signal. After incubation, the tubes were drained, and mussels were rinsed three times with FSW. Mussels were then fixed with 200 µl of 4% methanol-free formaldehyde (Pierce™, Thermo Fisher Scientific™).

### Image acquisition and analysis

#### Confocal microscopy

Samples were imaged within 24 h of staining using a flat-bottomed 12-well plate. Ten juvenile mussels were photographed from each sample. Mussels were placed in each well over a drop of gel (Tissue-Tek, O.C.T. Compound) to prevent the movement of the mussels and ensure they remained isolated during the imaging process.

The plate was viewed on an Olympus IX83 microscope equipped with a laser scanning confocal head FV3000 and a 4X N.A 0.16 objective. Samples were observed using a 405, 488 and 640 nm laser line, using the three detectors configured with a band pass filter of 410/20 to detect the autofluorescence of the shell (CH1; blue channel), 510/20 to detect the ROS-activated stain (CH2; green channel) and 660/20 to detect the auto-fluorescent signal from the digestive organs and ingested algae (CH3; red channel). The use of CH3 is optional as this channel is not used for image analysis to determine the amount of ROS in the samples but highlights the digestive tract of the mussels, which could be used as an indicator of the nutritional status of the mussels. The pinhole of the confocal head was extended to the maximum optical sectioning to improve signal-to-noise ratio and to reduce acquisition time. Acquisition settings were optimised using unstained samples with minimal positive signal. The equipment was set up using unstained samples to ensure the fluorescent signal from the shell obtained from CH1 and CH2 had the same level, with the intention to remove the unspecific signal from CH2 during the analysis. Each mussel was imaged on an axial dimension (350 µm) to cover the full thickness of the individuals. The original images were tailored for colour blindness, showing in colours magenta, green and cyan/turquoise for CH1, CH2 and CH3, respectively.

#### Image analysis

Images obtained using confocal microscopy were analysed using the software Fiji (Schindelin et al., 2012) and CellProfiler (Carpenter et al., 2006).

A z-projection of each image was performed and then processed to show maximum projection in three different channels (Fig. 3A). The signal from the shell of the mussel (CH1; blue channel) and the specific ROS signal (CH2; green channel) were then isolated (Figs 3B and 3C, respectively). A channel subtraction operation was then performed to remove any non-specific signal from the target channel (ROS; CH2) (Fig. 3D). Once the target channel (ROS) was clean, a ‘region of interest’ was applied to select the dimensions of the shell of the mussel from CH1 (Fig. 3E). The same selection was then applied to the image from CH2 (Fig. 3F). Hence, the area of ROS-positive signal inside the shell selection was determined, allowing quantification of ROS-signal to be standardised by the projected shell area.

### Statistical analysis

Proportional data for the ROS signal were transformed (arcsine square-root) prior to analysis. Assumptions of normality and homoscedasticity were checked using Shapiro­­–Wilk and Brown–Forsythe tests, respectively. ROS-positive area data were then analysed using a nested one-way ANOVA test using container as a nested random factor within treatment. Data were analysed using RStudio version 1.4.1717.
